# Single-cell characterization of the CD8^+^ T cell landscape and repertoire in HIV-1 Infection following antiretroviral therapy

**DOI:** 10.1186/s12985-026-03212-3

**Published:** 2026-06-07

**Authors:** Jun Lei, Jialingfang, Aifang Xu

**Affiliations:** 1 Department of Laboratory Medicine, Hangzhou Xixi Hospital, Zhejiang Chinese Medical University, Hangzhou, China; 2 Liangzhu Laboratory, Zhejiang University, Hangzhou, Zhejiang China; 3 Bone Marrow Transplantation Center of the First Affiliated Hospital, Center for Stem Cell and Regenerative Medicine, Zhejiang University School of Medicine, Hangzhou, Zhejiang China

## Abstract

Although antiretroviral therapy (ART) effectively suppresses HIV-1 viremia, immune dysfunction often persists. Here, we integrated single-cell RNA and T cell receptor (TCR) sequencing data to delineate the alterations of CD8⁺ T cells. Based on cross-sectional samples from different individuals (healthy donors, treatment-naïve individuals, and ART-treated individuals), we found ART promoted the transition of CD8⁺ T cells from exhausted toward naïve and memory-like subsets. Weighted gene co-expression network analysis identified distinct modules, including three (SM2-SM4) highly expressed in treatment-naïve individuals and one (SM6) significantly elevated post-ART. The reduced TCR diversity and increased clonality observed post-infection were not fully restored by ART, although ART partly altered clonal structure and CDR3 length. We established a predictive model based on the TCR features of CD8⁺ T cells, which accurately distinguished healthy donors, treatment-naïve individuals, and ART-treated individuals. These findings clarify the complex landscape of CD8⁺ T cell reconstitution, and identify key transcriptional networks and clonal dynamics during ART.

**Supplementary Information:**

The online version contains supplementary material available at 10.1186/s12985-026-03212-3.

## Introduction

The human immunodeficiency virus type 1 (HIV-1) continues to be a significant global public health issue, impacting millions of people. Without treatment, HIV-1 infection leads to a progressive decline in CD4^+^ T cells, progresses to acquired immunodeficiency syndrome (AIDS) [1–5], increases the risk of opportunistic infections, and ultimately leads to death [6, 7]. Antiretroviral therapy (ART) effectively reduces HIV-1 plasma viral load to undetectable levels in clinical tests. Although ART does not eradicate infected cells or halt viral protein production by infected cells, it can transform HIV-1 infection into a manageable chronic condition rather than a fatal disease, with early initiation and consistent adherence dramatically improving life expectancy [8–13]. Despite significant progress in ART, some challenges remain, such as chronic immune activation and immune exhaustion [14]. Chronic HIV infection drives sustained immune activation and upregulated PD-1/PD-L1 expression on T and B cells, promoting immune dysfunction and viral persistence [15]. Although early HIV-1 infection elicits robust T cell responses, chronic antigen exposure drives CD8^+^ T cell dysfunction and exhaustion [16, 17]. Approximately 30% of people living with HIV-1 on ART achieve viral suppression but remain immunological non-responders (INRs) due to inadequate CD4^+^ T cells recovery, which increases the risk of AIDS progression and related complication [18–22].

CD8^+^ T cells play a vital role in controlling HIV-1 replication by eliminating infected cells through their effector mechanisms [23–27]. The elimination of HIV-infected cells by CD8^+^ T cells is initiated by T cell receptor (TCR) recognition of viral peptides presented by MHC-I molecules. This recognition triggers T cell activation, which is further augmented by co-stimulatory signals from other immune cells and cytokines. Ultimately, the activated T cells selectively release cytotoxic granules containing perforin and granzymes to kill infected cells [28–30]. HIV-1 exhibits a high mutation rate, which facilitates immune evasion by enabling the virus to escape recognition by CD8⁺ T cells [31–33]. However, dual-reactive CD8^+^ T cells, which can recognize viral escape mutants, are present in some HIV-1-infected individuals who exhibit better control of the virus [34]. In our previous studies, we found the dual-reactive TCR exhibited a high affinity to recognize TL9 escape mutants presented by different HLA molecules. Interestingly, CDR3β of dual-reactive TCR does not contact antigen at all, but intensively recognizes the HLA only [35, 36]. Given the important role of CD8⁺ T cells in controlling HIV-1 infection, understanding the phenotypic and functional alterations of CD8⁺ T cells in HIV-1-infected individuals following ART is crucial for elucidating their functional roles in host antiviral defense.

Single-cell RNA and TCR sequencing provides high-resolution landscape of T cells, offering crucial insights into the immune system of HIV-1 infection [37–40]. Here, we performed integrated single-cell RNA and TCR sequencing analyses of public datasets to systematically analyze the phenotypic landscape, transcriptional programs, and clonal repertoire of CD8⁺ T cells in healthy donors (HD), treatment-naïve (TN), and ART-treated individuals (ART). It should be noted that the main comparison among the three groups is based on cross-sectional samples from different individuals. Thus, our findings reflect the correlation between CD8⁺ T cell characteristics and ART status, as well as the status of CD8⁺ T cells under continuous ART administration, rather than longitudinal dynamic changes during the initiation and progression of ART. We delineated subset shifts and treatment-associated gene modules, quantified ART-induced changes in the TCR repertoire, and built a TCR-based classifier to distinguish clinical groups.

## Methods

### Data source

The data that support the findings of this study are openly available in. Genome Sequence Archive of the Beijing Institute of Genomics (BIG) Data. Center, Chinese Academy of Sciences at http://bigd.big.ac.cn/gsa-human under reference number HRA000190. HRA000190 contains 4 HD samples, 9 TN samples and 8 ART samples. A total of 21 participants in this study, including HD, TN and ART. HD group: Four healthy participants (HD01-HD04), without HIV infection. TN group: Nine untreated HIV-infected participants (TN01-TN09) were included. All participants were HIV-positive without ART treatment, with detectable plasma viral load and baseline CD4⁺/CD8⁺ T cell counts recorded individually in Supplementary Table S1. Among them, TN07, TN08 and TN09 were paired participants, who were also sampled after ART treatment. ART group: Eight HIV-infected participants received standardized ART treatment. All maintained undetectable plasma viral load at the time of sampling. ART07, ART08 and ART09 corresponded to the paired longitudinal samples of TN07, TN08 and TN09, respectively. All individual demographic information, viral load, lymphocyte counts, treatment duration and medication regimens for each participant are listed in detail in Supplementary Table S1.

### Single-cell RNA and TCR sequencing data processing

The single-cell RNA-seq data was processed using the Seurat package [41]. Cells with low quality were removed. Specifically, the cells that expressing fewer than 100 genes or expressing more than 20% of mitochondrial genes were excluded to remove low quality cells. All single-cell data processing and downstream analyses were performed in R using the following packages: Seurat for quality control, normalization, dimensionality reduction, and clustering; dplyr for data manipulation; and patchwork for figure visualization.

The exhaustion score and cytotoxicity score were calculated using the AddModuleScore function in Seurat (v4.x) as previously described [41]. Briefly, the cytotoxicity score was defined by the mean expression levels of 12 established cytotoxic markers (PRF1, IFNG, GNLY, NKG7, GZMB, GZMA, GZMH, KLRK1, KLRB1, KLRD1, CTSW, and CST7), which represent key effector molecules involved in target cell killing and natural killer (NK) cell. The exhaustion score was calculated based on the expression of 7 well-characterized inhibitory receptors and transcription factors (LAG3, TIGIT, PDCD1, CTLA4, HAVCR2, TOX, and CD244), representing canonical T cell/NK cell exhaustion markers.

For each cell, the module score was computed by: (1) calculating the average expression of each signature gene set on the log-normalized data (RNA assay), (2) subtracting the aggregated expression of control feature sets (randomly selected from the same expression bin to account for cell-cell technical variation), and (3) centering the resulting scores across all cells. The resulting scores were visualized to depict the functional states of individual cells. The scTCR-seq data were processed with STARTRAC to analyze T cell dynamics [42]. The STARTRAC analysis provided quantitative single-cell TCR information, including the composition of T cells of distinct clone sizes, the state transition of T cells and clonal expansion.

### Weighted gene co-expression network analysis (WGCNA)

To identify gene co-expression modules associated with CD8^+^ T cell functional states, we performed WGCNA as previously described [43].

### Gene selection criteria

Genes were selected from the full gene set detected in scRNA-seq data, using the SetupForWGCNA function with the following criteria: (1) genes expressed in at least 5% of all CD8⁺ T cells (fraction = 0.05); (2) only protein-coding genes were retained; (3) mitochondrial and ribosomal genes were excluded to reduce technical noise. This filtering was applied to all genes from the entire scRNA-seq dataset to ensure robust and biologically meaningful co-expression patterns.

### Metacell construction

To reduce technical noise and computational burden while preserving biological heterogeneity, we constructed metacells by grouping cells based on cell type and condition (wgcna_para = cell_type_manual + condition_id). Metacells were generated using k-nearest neighbors (k = 10) with a maximum of 10 shared cells between metacells and a minimum of 10 cells per metacell group. The metacell expression matrix was normalized using log-normalization prior to network construction.

### Network construction and module detection

Co-expression networks were constructed using the signed network approach with a soft-thresholding power of 9, selected based on scale-free topology fitting (R² > 0.8). The topological overlap matrix (TOM) was calculated to measure gene interconnectedness, and hierarchical clustering was performed to identify gene modules using the dynamic tree cut algorithm with a minimum module size of minimum module size of 30 genes. Modules with highly correlated eigengenes (correlation > 0.75) were merged to ensure module independence.

### Module classification and characterization

All identified co-expression modules were termed SM (Single-cell Module). The prefix “S” indicates that these modules were derived from single-cell WGCNA (hdWGCNA), which is distinct from conventional bulk-based WGCNA modules. There are 9 modules that were selected (excluding the grey module containing unassigned genes) for future research. These modules were classified and characterized as follows.1. Module eigengenes (MEs): The first principal component of each module’s expression matrix was calculated as the module eigengene, representing the overall expression pattern of the module. 2. Hub gene identification: Intramodular connectivity (kME) was computed for each gene, defined as the correlation between gene expression and the module eigengene. The top 10 hub genes with highest kME values were identified for each module. 3. Functional annotation: Gene Ontology (GO) and KEGG pathway enrichment analyses were performed for each module using clusterProfiler (v4.x) to characterize the biological functions. Modules were named based on their dominant functional themes (e.g., cytotoxicity, exhaustion, interferon response) according to the enrichment results and hub gene identities. 4. Module-trait associations: Module eigengenes were correlated with sample metadata (cell type, condition, treatment response) to identify modules associated with specific biological states.

### Module visualization

Module relationships were visualized using eigengene dendrograms and correlation heatmaps. Intramodular gene networks were plotted for hub genes (*n* = 10 per module), with node size representing kME values and edge thickness representing topological overlap. Module UMAP embeddings were generated to visualize the relationships between modules in reduced dimensional space.

### Module network visualization

Module-specific gene networks were visualized using the ModuleNetworkPlot function in WGCNA [43]. For each module, the top 10 hub genes ranked by intramodular connectivity (kME) were positioned in the inner circle, while the remaining 15 genes (ranked 11–25 by kME) were placed in the outer circle.

### Edge definition and correlation measurement

Network edges represent gene-gene co-expression relationships quantified by the Topological Overlap Measure (TOM) [43]. The TOM between two genes was calculated as:

$$\rm TOM_{ij}=\frac {\sum_{ij} a_{iu}a_{uj}+a_{ij}}{min(k_{i},k_{j})+1-a_{ij}}$$

where is the adjacency matrix value (soft-thresholded Pearson correlation), and, are the connectivity measures for genes and, respectively [44]. TOM values range from 0 to 1, with higher values indicating stronger co-expression and shared neighbors. For visualization clarity, the top 500 strongest connections (sorted by TOM value) were displayed for each module. Edge transparency (alpha) was scaled by TOM strength to represent the strength of co-expression relationships.

### Network layout and topology

The circular network layout was generated using a force-directed algorithm (Fruchterman-Reingold) implemented in igraph. It is important to note that:1. The physical distance between nodes in the plot does not directly correspond to TOM values or correlation coefficients; 2. Each module network was plotted independently using the same layout algorithm, resulting in similar circular topographies across modules (SM2, SM3, SM4, SM6); 3. The differences between modules lie in the identity of the genes occupying the inner vs. outer circles and the specific gene-gene connections (edges), not in the overall network shape; 4. Node colors represent module assignment, and edge colors match the module color for intramodular connections.

### Pseudotime trajectory analysis

To reconstruct the differentiation trajectory of CD8^+^ T cells, we performed pseudotime analysis using Monocle 2 (v2.x) as previously described [45]. Briefly, CD8^+^ T cells were downsampled to a maximum of 200 cells per cell type per condition to balance computational efficiency and cell population representation. The Seurat object was converted to a CellDataSet object using the normalized count matrix. Highly variable genes (*n* = 2,000) identified by Seurat’s FindVariableFeatures function were used as ordering genes. Dimensionality reduction was performed using the DDRTree algorithm, and cells were ordered along the trajectory with State 2 designated as the root state based on the earliest pseudotime values and cellular identity. Based on the reconstructed trajectory topology, cells were assigned to three distinct cell fates: (1) “Pre-branch” (State 2), representing the root/starting population; (2) “Cell fate 1” (State 1), representing one terminal differentiation branch; and (3) “Cell fate 2” (States 3, 4, and 5), representing the alternative terminal differentiation branch. The two main functional cell fates (Cell fate 1 and Cell fate 2) were identified as the terminal branches diverging from the pre-branch state, representing distinct functional endpoints of CD8^+^ T cell differentiation. Differential gene expression analysis between cell fates was performed using differentialGeneTest in Monocle 2, and gene ontology enrichment analysis was conducted using clusterProfiler (v4.x) to characterize the functional properties of each fate. To identify genes with branch-specific expression patterns, we performed Branch Expression Analysis Modeling (BEAM) using the BEAM function with branch point 2 as the divergence point and the “duplicate” progenitor method. Genes with q-value < 1 × 10⁻⁴ were considered significantly branch-dependent and were visualized using branched heatmaps.

### TCR repertoire analysis

TCR repertoire metrics were calculated using VDJtools (v1.2.1) as previously described [44].

### Diversity and clonality metrics

TCR repertoire diversity was quantified using Shannon entropy index (H), calculated as:

$$\rm H=-\sum\;^{s}_{i=1}\,p_{i}\,In\, p_{i}$$

where represents the frequency of the *i*-th clonotype (defined by unique CDR3 amino acid sequence), and is the total number of unique clonotypes (richness).

Clonality was computed as normalized Shannon entropy to account for repertoire size variation:

$$\rm Clonality=1-\frac{H}{In(S)}$$

This metric ranges from 0 (perfectly uniform distribution, all clonotypes equally frequent) to 1 (complete clonal dominance, single clonotype). Richness was defined as the total number of unique CDR3 amino acid sequences detected per sample.

### Clonotype size classification

Clonotypes were categorized into five groups based on their cellular abundance: (1) single (1 cell), (2) small (2–5 cells), (3) medium (6–20 cells), (4) large (21–100 cells), and (5) hyperexpanded (> 100 cells).

### TCR clonotype dynamic analysis

To characterize the dynamics of TCR clonotypes before (TN) and after (ART), clonotypes were categorized into five groups based on their cellular abundance (TCR counts) in paired samples, with the following criteria: Expanded clones: Clonotypes present in both pre-ART and post-ART samples, with post-ART TCR count ≥ 2 × pre-ART TCR count. Contracted clones: Clonotypes present in both pre-ART and post-ART samples, with post-ART TCR count ≤ 0.5 × pre-ART TCR count. Eliminated clones: Clonotypes exclusively detected in pre-ART (TN) samples, with no detectable TCR counts in post-ART samples. Novel clones: Clonotypes exclusively detected in post-ART samples, with no detectable TCR counts in pre-ART (TN) samples. Persistent clones: Clonotypes present in both pre-ART and post-ART samples that did not meet the criteria for expanded, contracted, eliminated, or novel clones.

### Machine learning-based TCR classification and feature importance analysis

To classify TCR clonotypes based on CDR3 sequence features and identify discriminative molecular signatures, we developed a deep neural network (DNN) classifier and compared it with gradient boosting models.

### Feature engineering and selection

A total of 161 numerical features were extracted from CDR3 amino acid sequences, including: (1) physicochemical properties (hydrophobicity, molecular weight, isoelectric point, sequence length, average residue size); (2) amino acid composition frequencies (20 features); (3) V-gene and J-gene motif frequencies (40 features); and (4) positional encoding factors (p107-p113 Factor I-V, 60 features). Feature selection was performed in three steps: (1) features with > 50% missing values were excluded; (2) features with variance < 0.01 were removed using VarianceThreshold; (3) the top 60 features were selected based on mutual information scores using SelectKBest (mutual_info_classif). The final feature set was standardized (z-score) and missing values were imputed using median values.

### Deep neural network classifier

The DNN architecture consisted of four fully connected layers with batch normalization and dropout regularization: input layer (60 features) → 512 neurons (ReLU, dropout 0.4) → 256 neurons (ReLU, dropout 0.4) → 128 neurons (ReLU, dropout 0.3) → output layer (3 classes). The model was trained using cross-entropy loss with class weights to address imbalance, optimized with Adam (learning rate 1e-3, weight decay 1e-5). Learning rate scheduling combined linear warm-up (5 epochs) and cosine annealing (T_max = 50). Training used 5-fold stratified cross-validation with early stopping (patience = 10). The best model was selected based on validation accuracy.

### Feature importance analysis

Feature importance was assessed using two complementary approaches: (1) LightGBM gain importance: A LightGBM classifier (multiclass objective, 300 boosting rounds, early stopping) was trained on the same features, and gain-based importance scores were computed; (2) Neural network attribution: GradientSHAP (Gradient-based SHapley Additive exPlanations) was applied to the trained DNN using 200 background samples and 50 perturbations per feature to estimate SHAP values. The global importance was calculated as the mean absolute attribution across all samples.

#### Model evaluation

Classification performance was evaluated using accuracy, precision, recall, F1-score, and ROC-AUC. Confusion matrices were normalized by true class sizes to account for class imbalance.

## Results

### Single-cell RNA-seq analysis of CD8^+^ T cells in ART

**Fig. 1:**
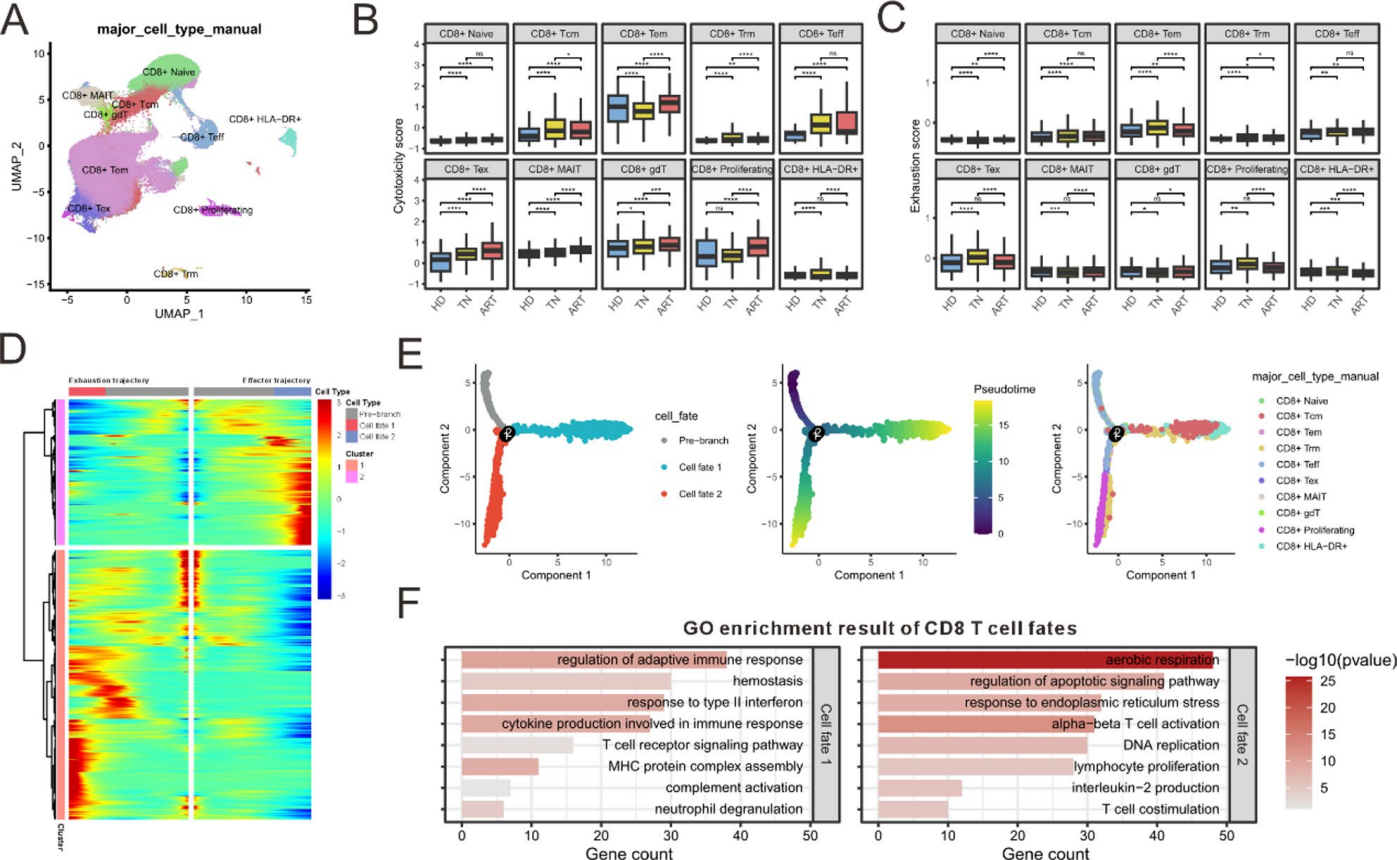
Single-cell transcriptomic profiling identifies altered heterogeneity and dysfunctional states of CD8^+^ T cells from all three clinical cohorts. (**A**) UMAP of CD8^+^ T cell subsets from HD, TN and ART, showing distinct distribution patterns of CD8^+^ T cell subsets. (**B**) The cytotoxicity scores from HD, TN and ART. Statistics were performed using a two-sided unpaired Wilcoxon test (*P < 0.05, **P < 0.01, ***P < 0.001, ****P < 0.0001). (**C**) The exhaustion scores from HD, TN and ART. Statistics were performed using a two-sided unpaired Wilcoxon test (*P < 0.05, **P < 0.01, ***P < 0.001, ****P < 0.0001). (**D, E**) Heatmaps showing the dynamic expression of key genes along the exhaustion trajectory and effector trajectory of CD8^+^ T cells, with analyses performed on all study samples. (**F)** GOBP enrichment analysis results for the exhaustion and effector trajectories of CD8^+^ T cells, with the top enriched biological processes listed

We collected the single-cell RNA/TCR-seq data from the published article to analyze the T-cell composition in HIV-1-infected individuals [38]. This study included 14 individuals with chronic HIV-1 infection and 4 healthy donors (HD) as controls. Among these individuals, 9 were TN and 8 were ART, among which 3 individuals had paired TN and ART samples. Based on the published single-cell sequencing data, we found that ART exhibited increased cytotoxicity and reduced exhaustion in both CD8⁺ T and CD4⁺ T cells compared to TN (Supplementary Fig. S1). Then, we identified 10 CD8⁺ T-cell subsets (Fig. 1A, Supplementary Fig. S2A) and 8 CD4⁺ T-cell subsets (Fig.S3A-B). In the CD8^+^ T-cell subsets analysis, we identified 10 clusters including CD8^+^ naïve T cells (CD8^+^ naïve), CD8^+^ central memory T cells (CD8^+^ Tcm), CD8^+^ effector memory T cells (CD8^+^ Tem), CD8^+^ tissue-resident memory T cells (CD8^+^ Trm), CD8^+^ effector T cells (CD8^+^ Teff), CD8^+^ exhausted T cells (CD8^+^ Tex), CD8^+^ mucosal-associated invariant T cells (CD8^+^ MAIT), CD8^+^ γδ T cells (CD8^+^ gdT), proliferating CD8^+^ T cells (CD8^+^ proliferating) and HLA-DR-positive CD8^+^ T cells (CD8^+^ HLA-DR^+^). In the CD4^+^ T-cell subsets analysis, we identified 8 clusters including CD4^+^ naïve T cells (CD4^+^ naïve), CD4⁺ T helper cells (CD4⁺ Th), CD4^+^ γδ T cells (CD4^+^ gdT), CD4^+^ mucosal-associated invariant T cells (CD4^+^ MAIT), CD4⁺ T memory T cells (CD4⁺ Tm), CD4⁺ cytotoxic T lymphocytes (CD4⁺ CTL), CD4⁺ regulatory T cells (CD4⁺ Treg) and proliferating CD4^+^ T cells (CD4^+^ proliferating). Compared to HD, TN showed an increase in CD8⁺ Tem, CD8⁺ Tex and CD8⁺ proliferating cells, while a decrease in CD8⁺ naïve, CD8⁺ Tcm and CD8⁺ MAIT cells (Supplementary Fig. S2B). In contrast, among CD4⁺ T cell subsets, CD4⁺ Tm, CD4⁺ CTL and CD4⁺ proliferating cells were increased, while CD4⁺ naïve T cells were reduced (Supplementary Fig. S3C). Compared to TN, ART showed an increase in CD8⁺ Naïve and CD8⁺ Tcm cells, while a decrease in CD8⁺ Tex and CD8⁺ Proliferating subsets (Supplementary Fig. S2B). In contrast, among CD4⁺ T cell subsets, CD4⁺ Proliferating cells were reduced (Supplementary Fig. S3C).

Compared with the TN, ART mainly elevated the cytotoxicity of CD8⁺ Tem, CD8⁺ Tex, CD8⁺ MAIT, CD8⁺ γδT and proliferating CD8⁺ T cells (Fig. 1B). Meanwhile, ART markedly reduced the exhaustion level in CD8⁺ Tem, CD8⁺ Tex, proliferating CD8⁺ T cells and HLA-DR⁺ CD8⁺ T subsets (Fig. 1C). In terms of CD4⁺ T cell subsets, relative to the TN group, ART elevated the cytotoxicity of CD4⁺ naïve T, CD4⁺ Th, CD4⁺ γδT, CD4⁺ Tm, CD4⁺ Treg, and proliferating CD4⁺ T cells (Supplementary Fig. S3D). Meanwhile, ART educed the exhaustion level in CD4⁺ Tm, CD4⁺ CTL, CD4⁺ Treg, and proliferating CD4⁺ T cells (Supplementary Fig. S3E).

Pseudotime trajectory analysis defines two differentiation paths of CD8⁺ T cells, which diverged into two main functional fates: one characterized by effector differentiation (Cell fate 1) and the other by exhaustion-related dysfunction (Cell fate 2) (Fig. 1E). Heatmap visualization of pseudotime-dependent gene expression captured the dynamic transcriptional changes underlying this transition (Fig. 1D). GO enrichment analysis further defined the biological functions of each fate. Cell fate 1 was enriched in adaptive immune response regulation, cytokine production, and T cell receptor signaling, while Cell fate 2 was associated with apoptotic pathway regulation, endoplasmic reticulum stress responses, DNA replication and lymphocyte proliferation (Fig. 1F).

### WGCNA identifies hub genes associated with ART

**Fig. 2:**
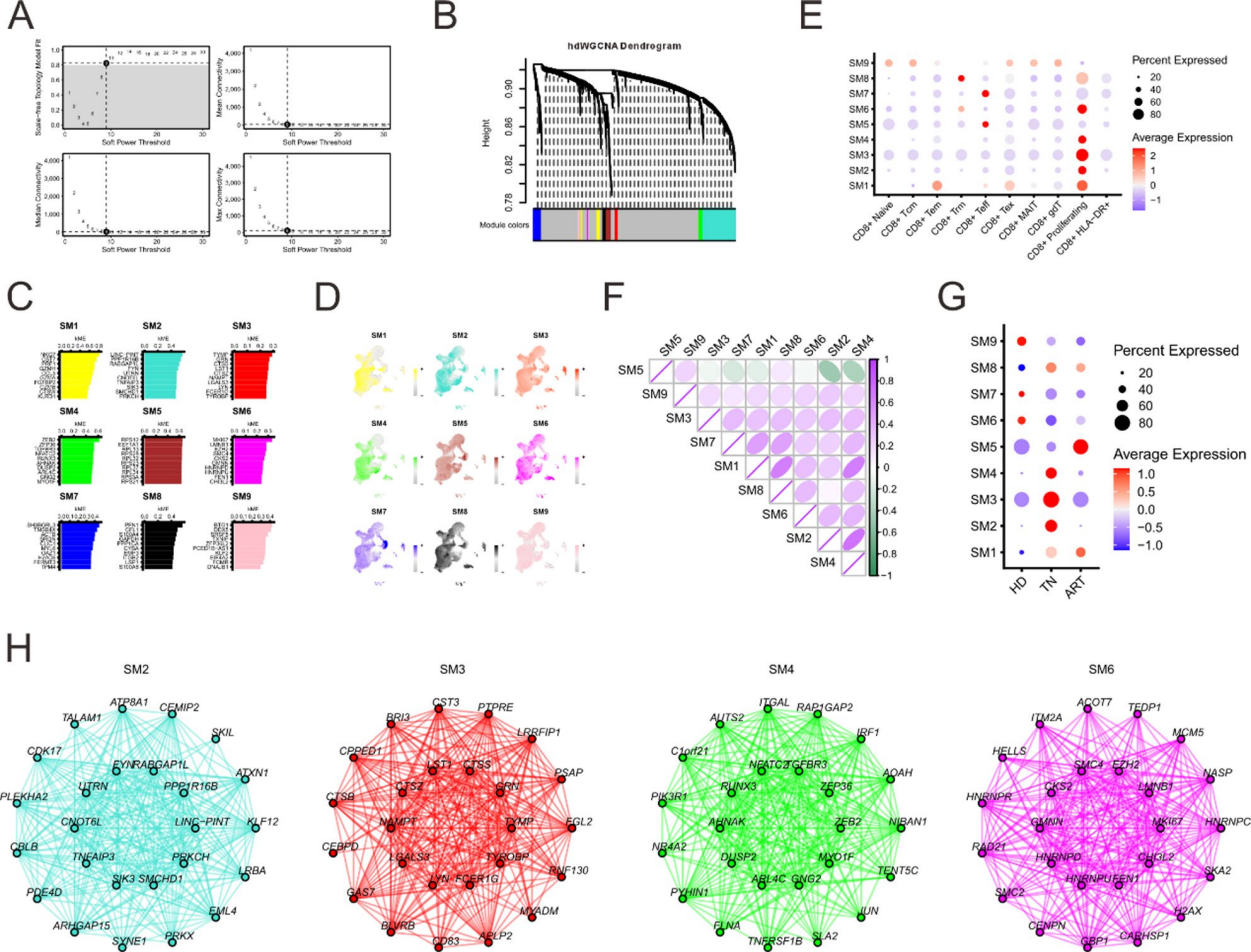
WGCNA co-expression network analysis uncovers key gene modules governing ART-induced CD8^+^ T cell functional alterations from all three clinical cohorts. (**A**) Scale-free topology model fit (R^2^) across varying soft power thresholds. The dashed line at R² = 0.8 indicates the threshold for acceptable scale-free topology, and the optimal power (9) is marked. (**B**) A total of nine modules were identified. (**C**) The top 10 genes within each module were identified. (**D**) The feather plot depicts the score for nine modules. (**E**) Bubble plot reveals the scores obtained of each module in CD8^+^ T cell subsets. (**F**) The dot plot indicating the expression features of gene module in HD, TN, and ART. (**G**) Bubble plot reveals the scores obtained of each module in HD, TN and ART. (**H**) Module networks of 4 hub modules

We performed the WGCNA algorithm to identify gene co-expression modules that characterize CD8⁺ T cell transcriptional programs in patients undergoing ART. We selected the optimal soft power threshold of 9 to constructed a co-expression network (Fig. 2A). Using this threshold, we identified 9 distinct co-expression modules (SM1-SM9) via hierarchical clustering (Fig. 2B). The top 10 hub genes in each module are shown in each module (Fig. 2C). The activity of each co-expression module across CD8⁺ T cells was visualized, revealing a distinct spatial distribution pattern for each module (Fig. 2D). These modules displayed subset-specific expression patterns across functionally distinct CD8⁺ T cell subsets. The CD8⁺ Proliferating exhibited the highest activity across nearly all modules (Fig. 2E). Module correlation analysis revealed both positive and negative regulatory relationships between these co-expression modules (Fig. 2F). Compared to HD, the SM2, SM3, SM4 module exhibited the highest module scores in the TN group, while ART exhibited similar module scores comparable to HD. In contrast, SM6 module scores was lowest in the TN group and elevated in ART (Fig. 2G). Network analysis of the core modules (SM2, SM3, SM4, SM6) further identified hub genes, which represent the key regulatory nodes in CD8⁺ T cell function during ART (Fig. 2H).

### ART partially alters the CD8^+^ T cell repertoire in HIV-1 infection

**Fig. 3:**
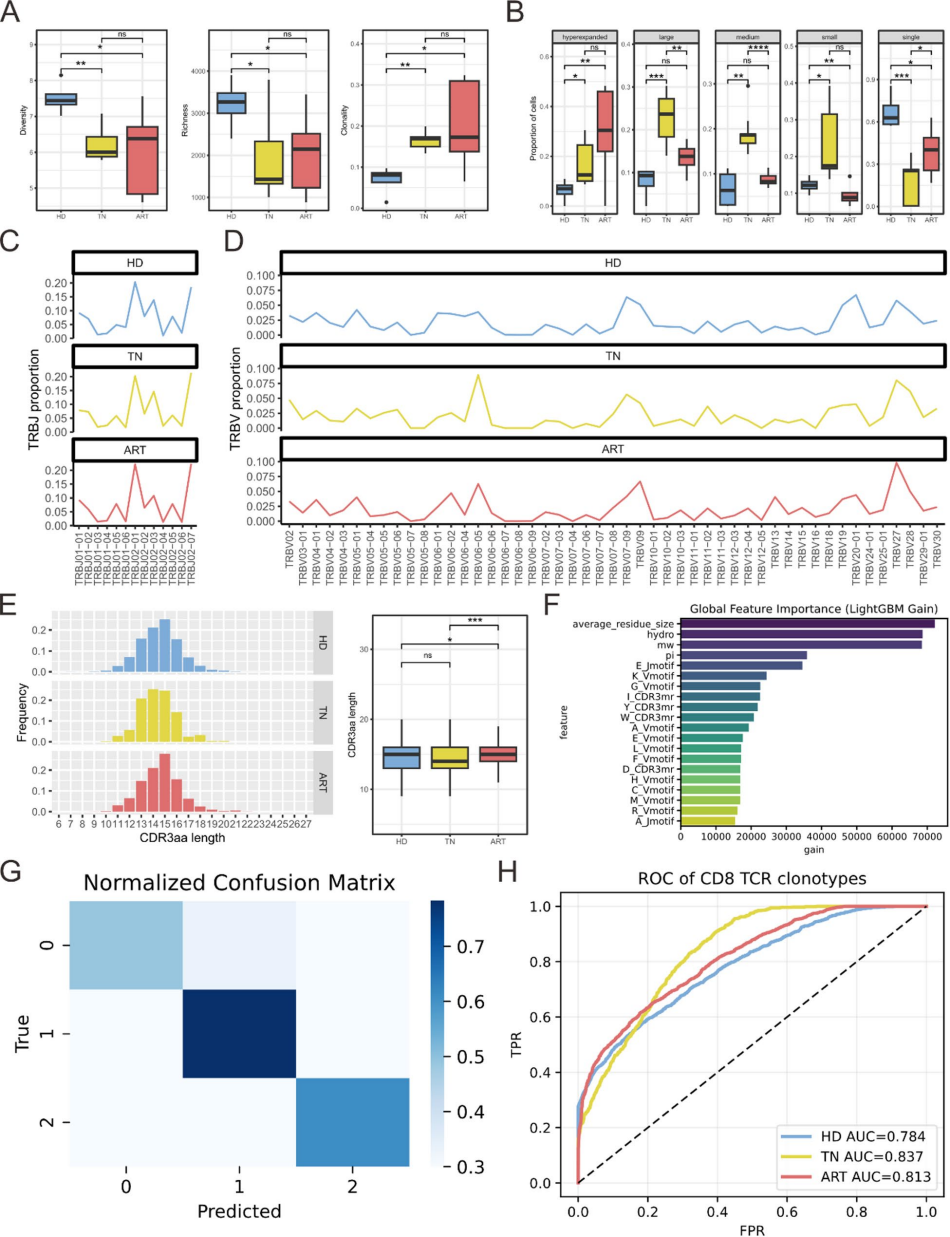
TCR repertoire profiling of CD8^+^ T cells reveals immune remodeling and predictive sequence signatures in ART. All analyses in this figure are based on CD8⁺ T cells from all three clinical cohorts (**A**) TCR repertoire characteristics of CD8^+^ T cells from HD, TN and ART groups, including clonality, diversity and richness. Statistics were performed using a two-sided unpaired Wilcoxon test (*P < 0.05, **P < 0.01). (**B**) Distribution of clonotype frequencies in the three groups, with statistical differences analyzed by two-sided unpaired Wilcoxon test (*P < 0.05, **P < 0.01, ***P < 0.001). (**C, D**) Line plots showing the usage frequency of TCR V and J genes from HD, TN and ART. (**E**) Distribution of TCR CDR3 amino acid lengths in HD, TN and ART, with box plots showing median, quartiles and outliers, and statistical significance assessed by two-sided unpaired Wilcoxon test (*P < 0.05, ***P < 0.001). (**F**) Global feature importance based on LightGBM model gain scores, showing the most predictive TCR properties. (**G**) Normalized confusion matrix of the classification model, where 0 = HD, 1 = TN, and 2 = ART. (**H**) Receiver operating characteristic (ROC) curves for distinguishing the three clinical groups, with the area under the curve (AUC) reported for each class

Then, we analyzed the CD8^+^ T cell repertoire in HD, TN and ART. Compared to HD, both TN and ART groups exhibited a decreasing trend in TCR diversity and richness of CD8⁺ T cells, along with increased clonality, although no significant difference was observed between TN and ART groups (Fig. 3A). Further subset-level comparison revealed heterogeneous TCR diversity across CD8⁺ T cell subsets. In line with the global trend of total CD8⁺ T cells, both naive CD8⁺ T cells and CD8⁺ Tcm cells showed reduced TCR diversity in the TN and ART groups compared with HD, and the diversity indexes were comparable between TN and ART. In contrast, compared with HD, CD8⁺ Tem, proliferating CD8⁺ T cells and CD8⁺ Tex cells in the TN group displayed significantly increased TCR diversity, while no significant changes were detected in the ART group. Of note, other specialized subsets, including CD8⁺ Trm, CD8⁺ Teff, CD8⁺ MAIT, CD8⁺ γδT and HLA-DR⁺ CD8⁺ T cells, maintained stable TCR diversity with no statistical differences among the three groups (Supplementary Fig. S4). Collectively, these subset-specific data demonstrate that HIV-1 infection and ART intervention drive heterogeneous remodeling of the CD8⁺ T cell TCR repertoire, which further complements our understanding of immune repertoire disturbance and immune reconstitution in chronic HIV infection. Further clonotype analysis revealed that the proportion of large and medium clones of CD8⁺T cells was lower in ART than in TN, while the single clones were higher (Fig. 3B). In addition, the amino acid length of the TCR CDR3 in CD8⁺T cells were shorter in TN but longer in ART (Fig. 3E). We next analyzed the TRBV and TRBJ gene usage in the three groups. The overall pattern of TRBJ gene usage was highly similar in the three groups (Fig. 3C). In contrast to TRBJ, TRBV gene usage showed obvious differences in the three groups. The HD exhibited a relatively balanced distribution, while TN displayed a highly skewed repertoire with prominent dominant peaks. Notably, ART showed partial restoration of TRBV usage balance, with attenuated clonal bias compared to TN (Fig. 3D).

Based on the sequence characteristics of the TCR CDR3 recognition region, we further constructed a predictive model incorporating TCR features of CD8⁺T cells such as average residue size, hydrophobicity, isoelectric point, V gene and J gene usage patterns. The top-ranked features included average residue size, hydrophobicity, and molecular weight (Fig. 3F), indicating that the physicochemical properties of the TCR sequence are the primary determinants distinguishing CD8⁺ TCR clonotypes across HD, TN, and ART. The normalized confusion matrix showed that the classification model achieved good performance in distinguishing TCR clonotypes from the three groups (Fig. 3G). Receiver operating characteristic (ROC) analysis was performed to evaluate the performance of the classification model for each group (Fig. 3H). The TN group showed the highest AUC value (0.837), followed by the ART group (0.813) and the HD group (0.784). This model can effectively distinguish among the HD, TN, and ART based on TCR characteristics of CD8⁺T cells. ART exhibited characteristics such as decreased clone size and increased CDR3 amino acid length. The predictive model established using multi-dimensional features of the TCR CDR3 region demonstrated good performance in distinguishing among HD, TN, and ART groups, suggesting that TCR characteristics may serve as potential indicators for assessing immune status.

We also analyzed the CD4^+^ T cell repertoire in HD, TN and ART. Compared to HD, both TN and ART groups exhibited a decreasing trend in TCR diversity and richness of CD4⁺T cells, while no significant change in clonality was observed. No significant differences were observed between TN and ART groups (Supplementary Fig. S5A). We further analyzed TRBV and TRBJ gene usage in CD4⁺ T cells. Similar to CD8⁺ T cells, the overall distribution of TRBJ gene usage was highly conserved among HD, TN, and ART (Supplementary Fig. 5C). The overall shape of the TRBV usage was broadly similar in the three groups (Supplementary Fig. 5D). We also constructed a predictive model incorporating TCR features of CD4⁺T cells, but this model did not effectively distinguish among the HD, TN, and ART based on TCR characteristics of CD4⁺T cells (Supplementary Fig. S5F-G).

### Functional diversification of reconstituting CD8^+^ T cell clones following ART

**Fig. 4:**
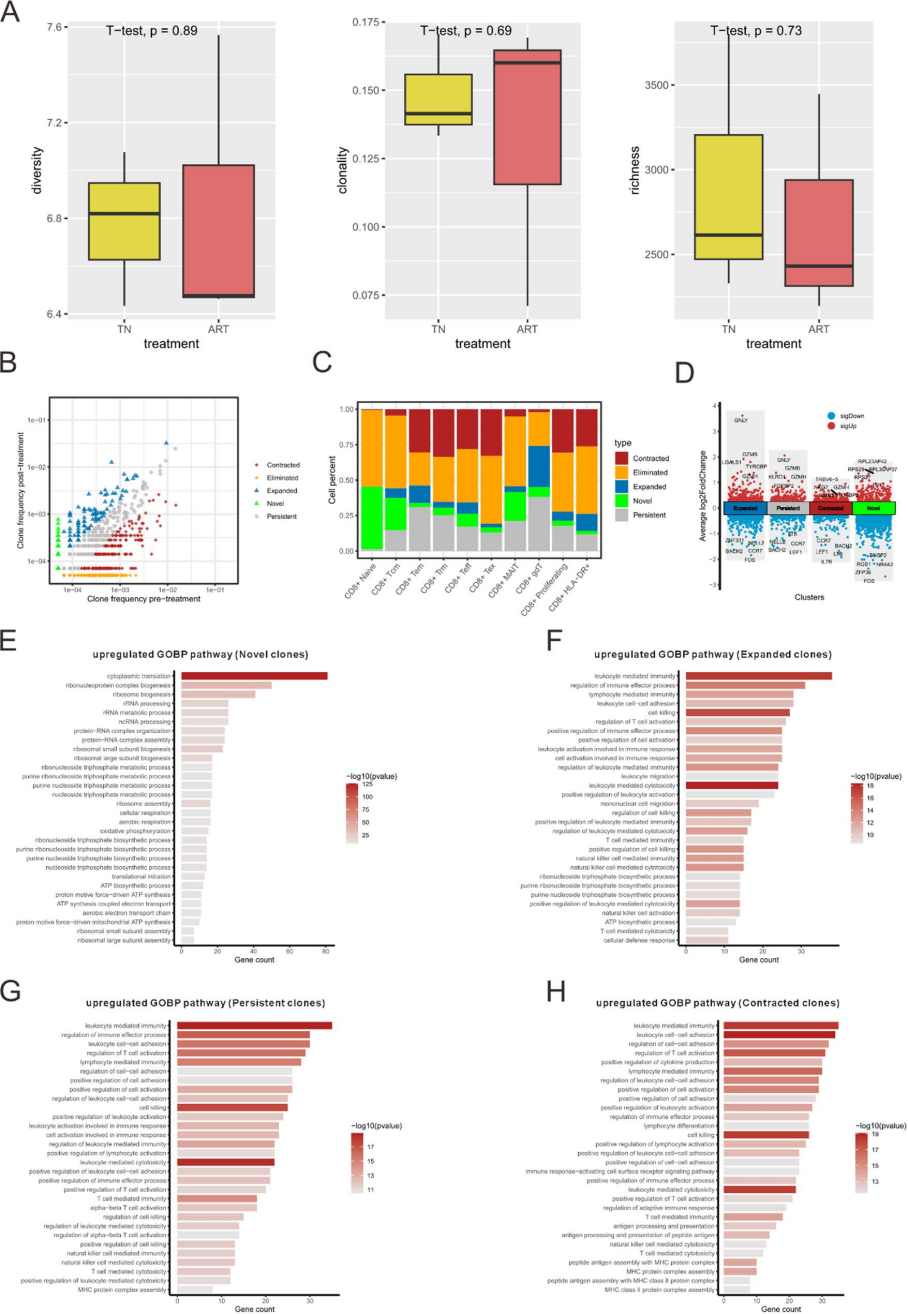
Clonal dynamics of CD8^+^ T cells demonstrates functional reprogramming of CD8^+^ T cells during ART. All analyses in this figure are based on paired longitudinal samples from three individuals with both pre-ART and post-ART (**A**) TCR repertoire comparison of CD8^+^ T cells pre- and post-ART. (**B**) Correlation analysis of clonotype sizes between pre- and post-ART samples, reflecting the dynamic changes of clonotypes before and after treatment. (**C**) Distribution of clonotypes in CD8^+^ T cell subsets post-ART. (**D**) The differential genes of novel, expanded, persistent, and contracted clones compared to the eliminated clones. (**E-H**) GOBP enrichment analysis of differential genes in novel, expanded, persistent and contracted clonotypes compared to eliminated clonotypes, with the top 30 enriched pathways listed for each clonotype, clearly indicating the functional differences between different clonotype subsets

To investigate the clonal dynamics of CD8⁺ T cells during ART, we analyzed paired pre- and post-treatment samples from three HIV-infected individuals (P07, P08, P09). Single-cell TCR analysis of CD8^+^ T cells from HIV-infected individuals before and after ART revealed no significant changes in TCR diversity, clonality and richness of CD8^+^ T cells post-treatment (Fig. 4A). To further investigate clonal dynamics, CD8^+^ T cells were categorized into five subsets based on TCR counts: novel clones, expanded clones, persistent clones, contracted clones and eliminated clones (Fig. 4B). To account for potential sampling bias, we note that clonotypes observed only once (classified as novel or eliminated) may not solely reflect true biological dynamics but could also be influenced by the sampling depth and sequencing sensitivity of our single-cell TCR assay. The clonal distribution of the CD8^+^ T cell subsets is shown in the Fig. 4C. Compared to eliminated clones, novel CD8^+^ T cells.

showed significant upregulation of pathways primarily related to cellular biosynthesis and metabolism, including cytoplasmic translation, ribosome biogenesis, rRNA processing, oxidative phosphorylation, and ATP biosynthetic processes (Fig. 4E). In contrast, expanded CD8^+^ T cells exhibited upregulation of immune effector pathways, such as leukocyte-mediated cytotoxicity, regulation of immune effector processes, T cell mediated immunity, and leukocyte activation (Fig. 4F). Similarly, persistent CD8^+^ T cells displayed enrichment in overlapping immune-related pathways, including leukocyte-mediated immunity, regulation of T cell activation, lymphocyte mediated immunity, and positive regulation of cell killing (Fig. 4G). The contracted CD8^+^ T cells also showed upregulation of immune pathways, but with a distinct focus on leukocyte cell-cell adhesion, regulation of T cell activation, MHC protein complex assembly, antigen processing and presentation, and positive regulation of lymphocyte activation (Fig. 4H).

## Discussion

Despite effective suppression of HIV-1 replication by ART, chronic immune dysfunction remains a major clinical obstacle in HIV-1 infection. Approximately 30% of individuals with HIV-1 infection, known as INRs, fail to restore functional CD4⁺ T cell counts and remain at higher risk for complications [18–22]. The mechanisms underlying this incomplete recovery are not fully understood. We found that CD8⁺ T cells shift from exhausted (Tex) and proliferating subsets towards naïve (Naïve) and central memory (Tcm) cells—is based on cross-sectional samples (different patients at different time points) rather than longitudinal samples (Supplementary Fig. S2B). This phenotypic rebalancing of the CD8⁺ T cells suggest partial restoration of CD8⁺ T cell homeostasis associated with the presence of ART. We acknowledge two key limitations of this study: first, we have not clarified whether this subset alteration exists during ART initiation and progression; second, we have not validated our main conclusion using longitudinal samples.

We also found that the proportion of CD8⁺ Tem was significantly higher in TN individuals compared to HD, and moderately reduced in ART (though not reaching statistical significance). This phenotypic change may be associated with the alterations of immune microenvironment induced by HIV-1 infection and ART intervention. CD8⁺ Tem is critical for mediating rapid antiviral immunity, as they can quickly differentiate into effector cells upon re-exposure to viral antigens. In TN, persistent HIV-1 replication and antigen stimulation drive the excessive expansion of CD8⁺ Tem cells. This expansion is a compensatory response of the immune system to control ongoing viral replication, which is consistent with the elevated exhaustion and cytotoxicity phenotypes of CD8⁺ T cells in TN individuals (Fig. 1B-C, supplementary S3D-E). In contrast, long-term suppressive ART effectively reduces viral load and antigen stimulation, leading to a moderate decline in the proportion of CD8⁺ Tem cells in ART. This reduction, together with the increased proportion of CD8⁺ naïve and CD8⁺ Tcm observed in our study, reflects a partial restoration of CD8⁺ T cell homeostasis after ART. It suggests that the immune system shifts from a sustained effector state (CD8⁺ Tem) toward a more quiescent, memory-predominant state (CD8⁺ naïve and CD8⁺ Tcm), which is conducive to maintaining long-term immune surveillance against HIV-1. Co-expression network analysis via WGCNA identified gene modules with distinct activity patterns across clinical groups. Modules SM2-SM4 exhibited the highest module scores in TN, whereas module SM6 showed a unique trend: its score was lowest in TN and elevated after ART, indicating partial recovery, with highest levels observed in HD. The hub genes within these modules may be high-value candidates for future mechanistic studies in modulating CD8⁺ T cell function.

An important finding of this study is the persistent alteration of the TCR repertoire in CD8⁺ T cells despite effective ART. Notably, ART failed to restore TCR diversity and clonality to the levels observed in HD. Although ART did not fully restore TCR repertoire structure, it induced smaller clone sizes and longer CDR3 regions in CD8⁺ T cells. Importantly, we built a TCR-based predictive model that accurately distinguishes clinical groups, indicating that the CD8⁺ TCR repertoire contains a quantifiable signature of disease and treatment status. Among all TCR features, average residue size was identified as the most important predictive variable. Average residue size represents the mean molecular volume of amino acids within the TCR CDR3β loop, which shapes the steric configuration and binding interface of the TCR. In TN, persistent viral antigen stimulation imposes strong selective pressure on CDR3 structure, leading to a characteristic pattern of average residue size. After long-term ART, relieved antigenic stress remodels the CDR3 structural property, shifting average residue size toward a profile closer to healthy donors. This structural change suggests average residue size serves as a sensitive structural marker reflecting immune reconstitution during ART. Notably, our study establishes a direct link between clonal fate and cellular function. Our data reveal that novel clones are enriched for metabolic and biosynthetic pathways, while expanded and persistent clones exhibit strong cytotoxic and immune effector signatures. We speculate that the newly recruited clones prioritize metabolic and biosynthetic pathways to support their proliferation and establishment, while the pre-existing, likely antigen-experienced clones remain in an effector state for rapid re-activation. The unique functional signature of contracted clones, emphasizing cell adhesion, immune regulation, and antigen presentation, may denote a transitional state or a subset with specialized regulatory functions. These data suggest that ART-mediated immune reconstitution relies on functionally distinct clonal T-cell subsets. The novel clones support cell metabolism, while the pre‑existing expanded clones maintain cytotoxic antiviral activity. Although global TCR repertoire remained stable during ART, our clonal fate classification demonstrates that profound functional reprogramming still occurs at clonal level. Such clonal functional diversification highlights that ART remodels the composition and biological function of T cell clones rather than simply restoring overall repertoire diversity. This clonal-level functional partitioning provides a refined mechanistic insight into how CD8⁺ T cell immunity is reconstructed and maintained during long-term suppressive ART.

Although our study comprehensively profiles transcriptomic heterogeneity and TCR clonal dynamics of CD8⁺ T cells during HIV infection and ART treatment, direct antigen-specific validation is absent in the current analysis. Despite this limitation, the robust clonal turnover and functional divergence across distinct clonal fates observed in paired samples indirectly reflect persistent antigen-driven selection pressure. Chronic HIV-1 antigen stimulation drives the accumulation of exhausted dominant clones in untreated individuals, whereas ART-mediated viral suppression reduces antigenic burden, facilitating clonal contraction, clonal renewal, and functional reprogramming of CD8⁺ T cells.

Furthermore, these findings advance our understanding of residual immune deficiency and HIV-1 persistence under suppressive ART. The incomplete restoration of TCR repertoire and retained clonal imprinting after treatment illustrates one key immunological barrier preventing full immune recovery. The persistent residual dysfunction of CD8⁺ T cells may contribute to sustained low-grade immune activation and HIV-1 reservoir maintenance. The functional diversification of reconstituting CD8^+^ T cell clones following ART indicates that future HIV-1 cure-oriented strategies, including therapeutic vaccination and immune checkpoint intervention, require targeted modulation of distinct clonal subsets to optimize antiviral immunity and promote more complete immune reconstitution. Importantly, the categorization of novel and eliminated clonotypes in is subject to limitations related to sampling depth and single-cell TCR sequencing sensitivity. Clonotypes detected only once at either pre- or post-treatment time point may represent undersampled low-frequency clones rather than true clonal loss or de novo expansion. The interpretation of novel/eliminated clonotypes should remain cautious. Future studies with larger cell numbers and deeper sequencing would help validate these observations.

In conclusion, our integrated multi-omics analysis delineates the complex and multifaceted adaptation of CD8⁺ T cells under ART. The identified transcriptional networks, the persistent TCR repertoire signature, and the model of clonal functional diversification provide a valuable framework for future research. Future studies in larger longitudinal cohorts, especially in INRs, will help establish these TCR and transcriptional signatures as predictive biomarkers for immune recovery.

## Supplementary Information

Below is the link to the electronic supplementary material.

Supplementary Material 1.

Supplementary Material 2.

## Data Availability

All data in this study supporting the findings are available upon request.
