# Supplementary material for: Single-cell characterization of the CD8+ T cell landscape and repertoire in HIV-1 Infection following antiretroviral therapy
Source: Virol J. 2026 Jun 7;23:191. doi: 10.1186/s12985-026-03212-3 (PMC13466494; doi:10.1186/s12985-026-03212-3)
Supplement: Supplementary file 1 [file 12985_2026_3212_MOESM1_ESM.docx]

**SUPPLEMENTARY INFORMATION**

**Single-cell characterization of** **the CD8^+^ T cell landscape and repertoire in HIV-1 Infection following antiretroviral therapy**

Jun Lei^1,†,*^, Jialingfang^2,3,†^, Aifang Xu^1^^,*^

^1^Department of Laboratory Medicine, Hangzhou Xixi Hospital, Zhejiang Chinese Medical University, Hangzhou, China.

^2^Liangzhu Laboratory, Zhejiang University, Hangzhou, Zhejiang, China.

^3^Bone Marrow Transplantation Center of the First Affiliated Hospital, and Center for Stem Cell and Regenerative Medicine, Zhejiang University School of Medicine, Hangzhou, Zhejiang, China.

^†^These authors contributed equally to this work.

^*^Corresponding authors. Email: [leijun@whu.edu.cn](mailto:leijun@whu.edu.cn); xuaifangxxh@163.com

**Supplementary Figures**

**Supplementary Figure S1**

**Figure S1.** **The exhaustion and cytotoxicity scores from HD, TN, and ART group.**

(A-B) The exhaustion and cytotoxicity scores of CD8^+^ T cells from HD, TN, and ART group. (C-D) The exhaustion and cytotoxicity scores of CD4^+^ T cells from HD, TN, and ART group. Statistics were performed using a two-sided unpaired Wilcoxon test (**P < 0.01, ****P < 0.0001).

**Supplementary Figure S2**

**Figure S2**. The single-cell transcriptomic profiling of CD8^+^ T cell subsets. (A) The dot plot indicates the expression of several marker genes in CD8^+^ T cell subsets. (B) Bar plots comparing the percentage of each CD4^+^ T cell subsets from HD, TN, and ART group. Statistics were performed using a two-sided unpaired Wilcoxon test (*P < 0.05, **P < 0.01, ***P < 0.001, ****P < 0.0001).

**Supplementary Figure S3**

**Figure S3**. Single-cell transcriptomic profiling identifies altered heterogeneity of CD4^+^ T cells from HD, TN and ART groups. (A) UMAP of CD4^+^ T cell subsets from HD, TN and ART, showing distinct distribution patterns of CD4^+^ T cell subsets. (B) The dot plot indicates the expression of several marker genes in CD4^+^ T cell subsets. (C) Bar graphs comparing the percentage of each CD4^+^ T cell subset from HD, TN and ART. Statistics were performed using a two-sided unpaired Wilcoxon test (*P < 0.05, **P < 0.01, ***P < 0.001, ****P < 0.0001). (D) The cytotoxicity scores from HD, TN and ART. Statistics were performed using a two-sided unpaired Wilcoxon test (*P < 0.05, **P < 0.01, ****P < 0.0001). (E) The exhaustion scores from HD, TN and ART. Statistics were performed using a two-sided unpaired Wilcoxon test (*P < 0.05, **P < 0.01, ***P < 0.001, ****P < 0.0001).

**Supplementary Figure S4**

**Figure S4**. TCR repertoire diversity of CD8⁺ T cell subsets from HD, TN and ART groups. Box plots showing the distribution of TCR diversity across 10 CD8⁺ T cell subsets in HD, TN and ART. Statistical significance between groups was assessed using the two-sided Wilcoxon rank-sum test. *P < 0.05, **P < 0.01, ***P < 0.001, ****P < 0.0001.

**Supplementary Figure S5**

**Figure S5**. TCR repertoire profiling of CD4^+^ T cells fail to distinguish HD, TN and ART. (A) TCR repertoire characteristics of CD4^+^ T cells from HD, TN and ART groups, including clonality, diversity and richness. Statistics were performed using a two-sided unpaired Wilcoxon test (*P < 0.05, **P < 0.01). (B) Distribution of clonotype frequencies in the three groups, with statistical differences analyzed by two-sided unpaired Wilcoxon test (*P < 0.05, **P < 0.01, ***P < 0.001). (C, D) Line plots showing the usage frequency of TCR V and J genes from HD, TN and ART. (E) Distribution of TCR CDR3 amino acid lengths in HD, TN and ART, with box plots showing median, quartiles and outliers, and statistical significance assessed by two-sided unpaired Wilcoxon test (*P < 0.05, ****P < 0.0001). (F) Normalized confusion matrix of the classification model, where 0 = HD, 1 = TN, and 2 = ART. (G) Receiver operating characteristic (ROC) curves for distinguishing the three clinical groups, with the area under the curve (AUC) reported for each class.
